# Prospective study of bilateral mix-and-match implantation of diffractive multifocal intraocular lenses in Koreans

**DOI:** 10.1186/s12886-018-0735-0

**Published:** 2018-03-07

**Authors:** Chan Min Yang, Dong Hui Lim, Sungsoon Hwang, Joo Hyun, Tae-Young Chung

**Affiliations:** 1 Department of Ophthalmology, Samsung Medical Center, Sungkyunkwan University School of Medicine, #81 Irwon-ro, Gangnam-gu, Seoul, 06351 South Korea; 2 Department of Preventive Medicine, Catholic University School of Medicine, Seoul, South Korea; 3 Department of Ophthalmology, Saevit Eye Hospital, Goyang, South Korea

## Abstract

**Background:**

To evaluate monocular and binocular visual outcomes for near, intermediate, and far distance in patients implanted with diffractive multifocal intraocular lenses (IOLs) with different add power contralaterally.

**Methods:**

This is a prospective contralateral study. Two diffractive multifocal IOLs with different added power were implanted bilaterally in twenty patients. TECNIS® ZKB00 (+ 2.75 D) was implanted in a dominant eye, and TECNIS® ZLB00 (+ 3.25 D) was implanted in a non-dominant eye. Uncorrected distance visual acuity (UDVA), uncorrected intermediate visual acuity (UIVA), uncorrected near visual acuity (UNVA), and manifest refraction (MR) values were measured at 1 month and 3 months postoperatively. At the 3-month follow-up, defocus curve, contrast sensitivity, and reading performance were evaluated. Quality of vision, overall satisfaction, and spectacle independence were evaluated by questionnaire.

**Results:**

Postoperative binocular UDVA, visual acuity at 80 cm, 60 cm, 50 cm, 43 cm, 33 cm were − 0.08 ± 0.10, 0.12 ± 0.14, 0.09 ± 0.09, 0.07 ± 0.11, 0.14 ± 0.09, 0.25 ± 0.11 logMAR. The binocular defocus curve showed an extended range of good visual acuity with sharp vision being observed from 0 D to − 2.50 D defocus (logMAR≤0.1). Reading performance was significantly improved compared to baseline. All patients were spectacle-free at distance, and 94.74% of the patients did not require glasses for near and intermediate vision.

**Conclusions:**

Mix-and-match implantation of diffractive multifocal IOLs with different add power provides an excellent wide range of vision, as well as high levels of visual quality and patient satisfaction.

**Trial registration:**

ClinicalTrials.gov, NCT02556944,

https://clinicaltrials.gov/show/NCT02556944

## Background

A monofocal intraocular lens (IOL) implanted after cataract extraction to replace the focusing power of the crystalline lens has a fixed focal length. Although patients can achieve a good uncorrected-distance visual acuity after monofocal IOL implantation, most patients need glasses for reading or other activities at close distance.

However, in recent years, the increasing use of smartphones and tablets and new leisure activities require a fast alternation of far and near distance tasks, also in elderly people. So spectacle dependence after cataract surgery can be inconvenient in the daily life of patients. In order to solve both cataract and presbyopia simultaneously, a variety of intraocular lenses have been developed. Diffractive bifocal IOLs with various levels of additional power have been widely used for correcting presbyopia after cataract surgery. The additional power of diffractive bifocal IOLs was selected according to patients’ lifestyle. Although diffractive bifocal IOL implantation is an effective way to satisfy patients who want to stop using their glasses after cataract surgery, it often results in visual symptoms, including diminished contrast sensitivity and dysphotopsia due to the IOLs’ diffractive surface [1, 2]. Other disadvantages of the implantation of bifocal IOLs is a suboptimal intermediate visual acuity compared to near and distance visual acuities [3, 4].

Diffractive trifocal IOLs aim to provide a wider range of spectacle independence especially at an intermediate distance compared to bifocal IOLs. Trifocal IOLs provide three foci to enhance intermediate visual acuity. However, the distribution of light energy for a third focus could negatively affect near and distance visual acuity [5]. Decreased contrast sensitivity and unwanted visual symptoms may also occur after trifocal IOLs implantation [6, 7].

Recently, several methods of combining different types IOLs have been introduced to meet the diverse needs of the patients [8, 9]. And bilateral mix-and-match implantation of diffractive multifocal IOLs with different add power may be another option for enhancing intermediate visual acuity. However, previous studies of contralateral implantation of diffractive multifocal IOLs with different add power have used AcrySof IQ ReSTOR [10, 11].

The purpose of this study was to evaluate the clinical outcomes following bilateral mix-and-match implantation of the recently developed Tecnis diffractive bifocal IOLs with + 2.75 and + 3.25 add power.

## Methods

This prospective, contralateral study comprised 20 patients affected by bilateral senile cataract. The study was approved by the Institutional Review Board of the Samsung Medical Center, and adhered to the tenets of the Declaration of Helsinki. Written informed consent was obtained from all patients.

The inclusion criteria were patients with bilateral senile cataract and the desire to be spectacle-free for all distances. Exclusion criteria were ages younger than 21 years, corneal astigmatism greater than 1.00 D, previous ocular surgery or trauma and ocular disease other than cataract. Hole-in-the card test was conducted in all patients for detection of dominant eye preoperatively.

The implanted IOLs were TECNIS ZKB00 (add power + 2.75 diopter [D], theoretical working distance 50 cm; Abbott Medical Optics, Santa Ana, California, USA) and TECNIS ZLB00 (add power + 3.25D, theoretical working distance 42 cm). The + 2.75D IOL was implanted in the dominant eye and that + 3.25D IOL in the non-dominant eye. Emmetropic intraocular lens power was selected from SRK/T, SRKII, Haigis, or Hoffer Q formulas according to corneal curvature, axial length and anterior chamber depth measured by IOLMaster version 5.4 (Carl Zeiss Meditec, Jena, Germany).

### Surgical technique

One experienced surgeon (T.Y.C) performed all surgical procedures under topical anesthesia using a standardized sutureless phacoemulsification with a 2.75 mm clear corneal incision. Steep axis corneal incision was created in eyes with corneal astigmatism of more than 0.5D, and temporal corneal incision was made in eyes with corneal astigmatism less than 0.5D. The non-dominant eye was operated first. After that contralateral surgery was performed at an interval of one week. Postoperative gatifloxacin and fluometholone 0.1% eye drops were used 4 times a day for 1 month.

### Patient evaluation

Preoperatively, all patients underwent a complete ophthalmologic examination including corrected and uncorrected visual acuity, manifest refraction, slit-lamp bio-microscopy, and fundus examination.

Patients were evaluated postoperatively at 1 day, 1 week, and 1 and 3 months. At 1 and 3 months after surgery, corrected and uncorrected visual acuity, manifest refraction, defocus curve, contrast sensitivity, reading performance, and subjective satisfaction were examined.

All patients underwent measurement of corrected and uncorrected distance visual acuity at 5 m (CDVA and UDVA). Uncorrected intermediate visual acuities (UIVA) were measured at 60 cm and 80 cm and uncorrected near visual acuities (UNVA) at 33 cm, 43 cm, and 50 cm using the ETDRS chart. All visual acuity were measured monocularly and binocularly.

Defocus curves were plotted by measuring the visual acuity under photopic condition at 5 m, adding lenses in 0.5D increments from − 4.0 to + 2.0D.

Contrast sensitivity at 3, 6, 12, and 18 cycles per degree was measured using a CSV-1000 chart (Vector Vision, Greenville, OH) under photopic (85 cd[cd]/m^2^) and mesopic (~ 3 cd/m^2^) conditions at 3 months after surgery. Results were converted in log units for statistical analysis using a specific table for the CSV-1000 [12].

At baseline and 3 months postoperatively, reading performance was measured using an iPad application at 50 cm [13]. The print size of the reading chart ranges from 1.0 to 0 logarithm of the minimal angle of resolution (logMAR). Average reading speed in words per minute (wpm) was calculated with the iPad application. Critical print size was defined as the last acuity measured before the reading speed was reduced below the 95% confidence interval of that individual’s average reading speed [14]. Threshold print size was determined as the smallest print size that could be read and expressed in logarithm of the reading acuity determination (logRAD).

One and three months after surgery, all patients were asked to complete the questionnaire regarding overall satisfaction, presence of visual artifacts, and dependency on spectacles for near, intermediate and far vision. Overall satisfaction was evaluated using 5 levels (very satisfied, satisfied, neither satisfied nor dissatisfied, unsatisfied, very unsatisfied). Severity of visual artifacts, divided into 4 levels (none, minimal, moderate and severe), were assessed using a Quality of Vision questionnaire [15]. Furthermore, patients were asked if they would choose the same IOL again.

### Statistical analysis

All data are presented as mean ± standard deviation. The statistical analysis was performed using SPSS software version 18.0 (SPSS, Inc., Chicago, IL). Measured decimal visual acuities were converted to logMAR for data analysis. Because the variables did not follow a normal distribution, non-parametric statistical analysis was used. The Wilcoxon signed-rank test was applied to assess the difference between preoperative and postoperative data. The Mann-Whitney U test was used to compare the dominant and non-dominant eyes. A sample size of 17 patients would allow the detection of a minimum clinical relevant difference in depth of focus with a standard deviation of 5.8. The sample sizes took into account a significance level of 5% and a power of 80% for a 2-sided test. Assuming an proportion of withdrawal of 10%, 20 patients were included.

## Results

A total of 20 patients were enrolled, of which 19 completed the study. Patient recruitment was from August 2015 to January 2016. The study was finished after 3 months postoperative follow-up visit was completed for all patients in April 2016. All patients received regular follow-up examinations for at least 3 months. The mean age was 60.1 ± 6.61 years (range: 45 to 70 years), 63.1% (12 of 19) of the patients were female. Preoperative mean axial length was 24.74 ± 1.43 mm (range: 22.19 mm to 27.87 mm), mean keratometric value was 43.18 ± 1.25 D (range: 40.91 D to 45.18 D). Preoperative mean anterior chamber depth was 3.15 ± 0.49 mm (range: 2.42 mm to 4.53 mm). The mean IOL power implanted was 18.5 ± 4.4 D (range: 7.5 D to 25.5 D). Table 1 shows preoperative and postoperative monocular refractive results and visual acuities. At 3 months, there were statistically significant improvements in CDVA, UDVA, UIVA, and UNVA (*p* < 0.001). However, UNVA at 33 cm of eye with ZKB00 was not significantly different compared to the preoperative value (*p* = 0.178). No significant differences between eyes implanted with ZKB00 and eyes implanted with ZLB00 were found in uncorrected and corrected visual acuity at all distances (*p* > 0.05).

**Table 1:** Monocular refractive results and visual acuities in patients implanted with ZKB00 and ZLB00 multifocal IOLs at preoperative, 1 month and 3 months after surgery

Measurement	Preoperative		1 month				3 months			
	Dominant eye	non-dominant eye	Dominant eye	*P* value^a^	non-dominant eye	*P* value^b^	Dominant eye	*P* value^c^	non-dominant eye	*P* value^d^
Spherical equivalent (D)	−1.61 ± 3.71	−1.69 ± 3.92	− 0.01 ± 0.32		0.00 ± 0.30		− 0.03 ± 0.22		0.06 ± 0.27	
CDVA at 5 m	0.09 ± 0.13	0.21 ± 0.24	− 0.04 ± 0.08	0.005	− 0.04 ± 0.07	< 0.001	−0.06 ± 0.07	< 0.001	−0.06 ± 0.07	< 0.001
UDVA at 5 m	0.53 ± 0.45	0.62 ± 0.45	− 0.03 ± 0.09	< 0.001	−0.02 ± 0.10	< 0.001	−0.02 ± 0.10	< 0.001	−0.01 ± 0.10	< 0.001
UIVA at 80 cm	0.61 ± 0.27	0.67 ± 0.32	0.26 ± 0.17	0.002	0.27 ± 0.16	0.001	0.19 ± 0.14	< 0.001	0.19 ± 0.12	< 0.001
UIVA at 60 cm	0.66 ± 0.15	0.66 ± 0.27	0.12 ± 0.12	0.003	0.18 ± 0.14	0.005	0.16 ± 0.12	0.003	0.25 ± 0.13	< 0.001
UNVA at 50 cm	0.62 ± 0.23	0.65 ± 0.26	0.13 ± 0.12	< 0.001	0.14 ± 0.12	< 0.001	0.14 ± 0.13	< 0.001	0.16 ± 0.12	< 0.001
UNVA at 43 cm	0.58 ± 0.21	0.69 ± 0.18	0.24 ± 0.15	< 0.001	0.22 ± 0.13	< 0.001	0.21 ± 0.11	< 0.001	0.16 ± 0.10	< 0.001
UNVA at 33 cm	0.46 ± 0.29	0.53 ± 0.31	0.41 ± 0.21	0.441	0.30 ± 0.20	0.005	0.36 ± 0.18	0.178	0.30 ± 0.13	0.008

CDVA Corrected distance visual acuity, UDVA Uncorrected distance visual acuity, UIVA Uncorrected intermediate visual acuity, UNVA Uncorrected near visual acuity

^a^Dominant eye: Preoperative to 1 month after surgery

^b^Nondominant eye: Preoperative to 1 month after surgery

^c^Dominant eye: Preoperative to 3 months after surgery

^d^Nondominant eye: Preoperative to 3 months after surgery

**Fig. 1:**
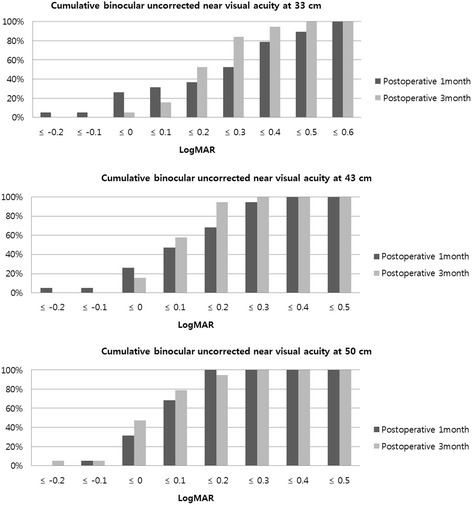
Postoperative cumulative binocular uncorrected near visual acuity (UNVA) in patients implanted with ZKB00 and ZLB00 multifocal intraocular lens at 33 cm, 43 cm, and 50 cm in logarithm of the minimal angle of resolution (logMAR)

**Fig. 2:**
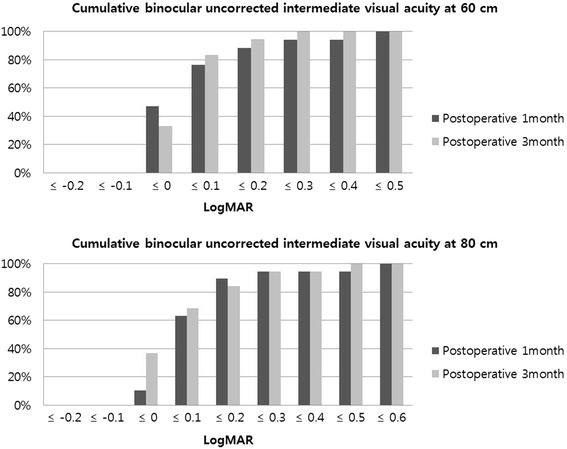
Postoperative cumulative binocular uncorrected intermediate visual acuity (UIVA) in patients implanted with ZKB00 and ZLB00 multifocal intraocular lens at 60 cm and 80 cm in logarithm of the minimal angle of resolution (logMAR)

**Fig. 3:**
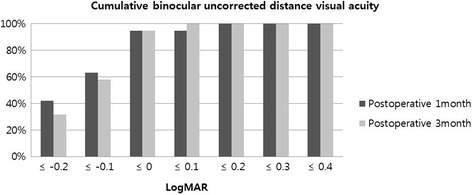
Postoperative cumulative binocular uncorrected distance visual acuity (UDVA) in patients implanted with ZKB00 and ZLB00 multifocal intraocular lens in logarithm of the minimal angle of resolution (logMAR)

Table 2 shows preoperative and postoperative binocular visual acuities. Postoperative binocular visual acuities were significantly better than preoperative values, except for binocular UNVA at 33 cm. Cumulative binocular UNVA, UIVA, and UDVA at 1 and 3 months after surgery are shown in Figs. 1, 2 and 3.

**Table 2:** Binocular visual acuities in patients implanted with ZKB00 and ZLB00 multifocal IOLs at preoperative, 1 month and 3 months after surgery

Measurements	Preoperative	1 month	*p* value^a^	3 months	*p* value^b^
CDVA at 5 m	0.03 ± 0.12	− 0.11 ± 0.08	0.001	−0.12 ± 0.08	0.001
UDVA at 5 m	0.36 ± 0.27	−0.10 ± 0.10	< 0.001	−0.08 ± 0.10	< 0.001
UIVA at 80 cm	0.50 ± 0.27	0.21 ± 0.23	0.013	0.12 ± 0.14	< 0.001
UIVA at 60 cm	0.51 ± 0.18	0.10 ± 0.14	0.003	0.09 ± 0.09	0.003
UNVA at 50 cm	0.43 ± 0.19	0.10 ± 0.10	< 0.001	0.07 ± 0.11	< 0.001
UNVA at 43 cm	0.45 ± 0.17	0.16 ± 0.15	< 0.001	0.14 ± 0.09	< 0.001
UNVA at 33 cm	0.31 ± 0.30	0.27 ± 0.23	0.491	0.25 ± 0.11	0.348

CDVA Corrected distance visual acuity, UDVA Uncorrected distance visual acuity, UIVA Uncorrected intermediate visual acuity, UNVA Uncorrected near visual acuity

^a^Preoperative to 1 month

^b^Preoperative to 3 months

**Fig. 4:**
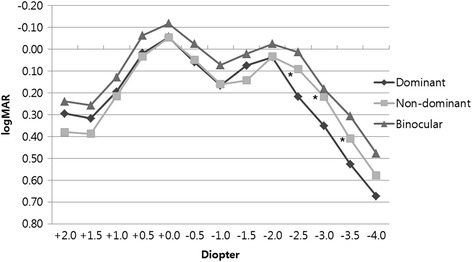
Monocular and binocular defocus curve plotted in logarithm of the minimal angle of resolution (logMAR) in patients implanted with ZKB00 and ZLB00 multifocal intraocular lens at 3 months postoperatively. (*: *p* < 0.05, between dominant and non-dominant eye)

Monocular and binocular defocus curves are shown in Fig. 4. Eyes implanted with ZKB00 and ZLB00 had two peaks at 0 and − 2 D. When comparing both eyes, ZKB00 eyes had a slightly better visual acuity from − 1.0 to − 2.0 D. However, these differences were not statistically significant (*p* = 0.84, *p* = 0.103 and *p* = 0.908, respectively). Eyes with ZLB00 showed significantly better visual acuity at − 2.5, − 3.0, and − 3.5D compared to eyes implanted with ZKB00 (*p* = 0.003, *p* = 0.022 and p = 0.022, respectively). The binocular defocus curve also showed two peaks and overlapping curves with monocular defocus curves, as well as a wider range of good visual acuity from 0 to − 3.0 D (log MAR < 0.2 [range; − 0 ~ − 3.0 D], logMAR< 0.1 [range: 0–2.5 D]) Eyes with ZKB00 had a slightly better visual acuity from − 1.0 to − 2.0 diopters (D) compared to eyes with ZLB00. Eyes with ZLB00 showed significantly better visual acuity at − 2.5, − 3.0, and − 3.5 D (Mann-Whitney U test, *p* values < 0.05). Binocular defocus curve showed good visual acuity better than 0.1 logMAR from 0 to − 2.5 D.

**Fig. 5:**
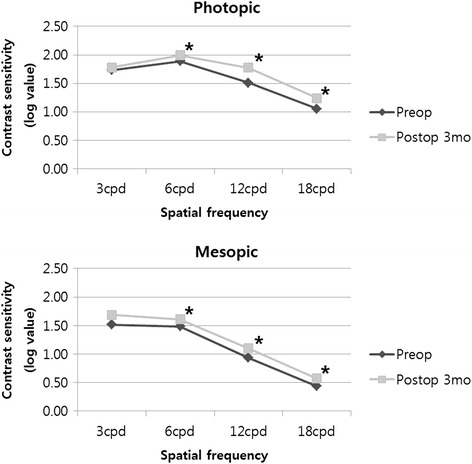
Mean binocular photopic (Top) and mesopic (Bottom) contrast sensitivity in patients implanted with ZKB00 and ZLB00 multifocal intraocular lens

Postoperative contrast sensitivity at 3 months was statistically significantly better at some spatial frequencies than that measured preoperatively (Fig. 5).

The mean reading speed increased from 76.93 ± 17.47 wpm (range: 44.33 to 106.17 wpm) at baseline to 86.83 ± 17.45 wpm (range: 64.99 to 123.53 wpm) at 3 months after surgery. The mean critical print size decreased from 0.65 ± 0.22 logRAD (range: 0.1 to 0.8 logRAD) at baseline to 0.24 ± 0.13 logRAD (range: 0 to 0.5 logRAD) at 3 months after surgery. The threshold print size also decreased from 0.35 ± 0.22 logRAD (range: 0 to 0.8 logRAD) at baseline to 0.14 ± 0.13 logRAD (range: 0 to 0.4 logRAD) at 3 months after surgery. There were statistically significant differences in mean reading speed, critical print size, and threshold print size between baseline and 3 months after surgery (*p* = 0.07, *p* = 0.01 and *p* = 0.03, respectively).

Overall satisfaction, visual symptoms and spectacle dependence are summarized in Table 3. Postoperative overall satisfaction with distance vision was statistically significant regarding the improvement achieved compared to preoperative (*p* < 0.05). Although halo and starburst were increased at 1 and 3 months after surgery, there were no significant differences compared to baseline (halo: *p* = 0.108 and *p* = 0.301, respectively; starburst: *p* = 0.890 and *p* = 0.209, respectively). All patients reported complete spectacle independence for distance after surgery. One patient required spectacles for near vision and another one patient sometimes for intermediate vision after surgery. A total of 94.7% of the patients (18 of 19) answered that they would choose the same IOLs again and 89.4% (17 of 19) did not feel dizzy and recognized the difference between both eyes.

**Table 3:** Overall satisfaction, visual artifact questionnaire response and spectacle dependence in patients implanted with ZKB00 and ZLB00 multifocal IOLs at preoperative, 1 month and 3 months after surgery

	Preoperative	1 month	*p* value^a^	3 months	*p* value^b^
Overall satisfaction					
Far	2.68 ± 0.89	4.37 ± 0.50	< 0.001	4.42 ± 0.51	0.001
Intermediate	2.68 ± 0.89	3.95 ± 1.13	0.003	4.11 ± 0.81	0.001
Near	2.84 ± 1.01	3.84 ± 0.90	0.013	3.89 ± 0.88	0.008
Visual artifact					
Glare	0.47 ± 1.02	0.58 ± 1.07	0.777	0.21 ± 0.71	0.334
Halo	0.47 ± 1.02	1.11 ± 1.33	0.108	0.84 ± 1.12	0.164
Starburst	0.37 ± 0.96	0.32 ± 0.75	0.890	0.79 ± 1.13	0.179
Spectacle use					
Far	73.68%	0.00%	< 0.001	0.00%	< 0.001
Intermediate	78.94%	5.26%	< 0.001	5.26%	< 0.001
Near	63.16%	5.26%	< 0.001	5.26%	< 0.001

Overall satisfaction: 1 = very dissatisfied; 2 = dissatisfied; 3 = neither satisfied nor dissatisfied; 4 = satisfied; 5 = very satisfied

Visual artifacts: 0 = none; 1 = minimal; 2 = moderate; 3 = severe

Visual artifact was assessed using the Quality of Vision questionnaire

^a^Preoperative to 1 month

^b^Preoperative to 3 months

## Discussion

In this prospective study, the clinical outcomes of mix-and-match implantations of ZKB00 and ZLB00 were showed good UCVA and UNVA as well as UIVA and high satisfaction without visual disturbance such as glare and halo. Although there was previous study comparing ZMB00, ZKB00 and ZLB00, we could confirm that depth of focus was increased through contralateral mix-and-match implantation of ZKB00 and ZLB00. Compared with previous studies using the trifocal diffractive IOLs, our results revealed that contrast sensitivity was not reduced and visual disturbance was less. The previous version, ZMB00 Tecnis multifocal IOLs with + 4.0 D add power has the same design as the studied IOLs; however, the study IOLs have a relatively lower add power of + 2.75 D and + 3.25 D. All IOLs of this platform have a refractive zone on the anterior surface to provide distance vision and a full diffractive posterior surface for near vision. The fewer diffractive rings of ZKB00 and ZLB00 compared to ZMB00 are considered to reduce unwanted visual symptoms [16]. Regardless of pupil size, the light is evenly distributed between distance and near foci. Other optical principles of multifocal IOLs are dependent on pupil size [17].

Other studies with ZKB00 and ZLB00 IOL implantation report that subjects implanted with low add power bifocal IOLs had good intermediate and distance visual acuity with a high level of satisfaction [16, 18, 19]. Kretz et al. [19] reported 63.3% of the patients implanted with ZKB00 in both eyes achieved a binocular UIVA at 80 cm of 0.1 logMAR or better. In this study, the percentage of patients with binocular logMAR UIVA better than 0.1 logMAR at 80 cm was 68.5%. Kretz et al. [18] reported that bilaterally implantation of the ZLB00 IOL revealed a binocular UIVA of 0.06 ± 0.09 logMAR at 60 cm. In our study, we found comparable results with 0.09 ± 0.09 logMAR. However, previous studies did not include the defocus curve which makes it difficult to compare the achieved visual acuity at various distances directly. Previous studies of bilateral mix-and-match implantation of diffractive bifocal IOLs reported a better visual acuity over a wider range compared to bilateral implantation of IOLs with the same add power [11]. Our study with mix-and-match implantations of Tecnis ZKB00 and ZLB00 found a 0.1 logMAR or better visual acuity in the 0 to − 2.5 D range of the defocus curve. We could speculate that outcomes of mix-and-match implantation of Tecnis ZKB00 and ZLB00 might be better visual acuity at a broader range than bilateral implantation of IOLs with the same add power (ZKB00 or ZLB00).

This study is the first prospective study applying the bilateral mix-and-match implantation of Tecnis ZKB00 and ZLB00. All previous studies on mix-and-match implantations of diffractive bifocal IOLs used the AcrySof ReSTOR IOL [10, 11]. Nakamura et al. [10] reported that contralateral implantation of ReSTOR IOLs with + 3.0 and + 4.0 D addition was an effective way to get a broad range of good uncorrected visual acuity in the defocus curve. Mastropasqua R et al. [11] also reported that patients, implanted with ReSTOR IOLs with contralateral + 2.5 and + 3.0 D additions, had good uncorrected visual acuity over a wide range, and contrast sensitivity and visual quality did not decrease compared to bilateral implantation of diffractive multifocal IOLs with the same additional power. Compared with the defocus curve of Mastropasqua et al., our study revealed 0.1 logMAR or better vision from 0 to − 2.5 D, whereas Mastropasqua et al. report 0.1 logMAR or better in the range from 0D and − 1.5 ~ − 2.5D. In the range of intermediated distance from − 0.5D to − 1.5D, the results of ours study appear better than those of Mastropasqua et al. Although the add power differs slightly between studies, it seems that the IOL design is responsible for the better intermediate vision. And it may be due to differences in clinical characteristics of patients, such as axial length that can affect effective lens position.

Recently, trifocal diffractive IOLs were developed to provide better intermediate visual acuity. So far, no direct comparative study between bilateral implantation of diffractive trifocal IOLs and contralateral implantation of diffractive bifocal IOLs has been published. Ours study shows 0.1 logMAR or better visual acuity in the range from 0 to − 2.5D in the defocus curve and it was comparable to or slightly better than that reported in previous studies on trifocal diffractive IOLs [20].

Multifocal IOLs had a drawback in decreasing contrast sensitivity However, for Tecnis multifocal IOLs it was known as the prolate anterior surface could improve the mesopic contrast sensitivity [17, 21]. Gierek-Ciaciura et al. [22] reported that eyes with ZM900 Tecnis multifocal IOLs had better contrast sensitivity than eyes with other diffractive multifocal IOLs or refractive multifocal IOLs. Kim et al. [16] found that contrast sensitivity was higher in subjects with ZKB00 or ZLB00 than subjects with ZM900. This study, using ZKB00 and ZLB00, also showed improvement of contrast sensitivity compared with preoperative contrast sensitivity, and statistically significant improvement in some spatial frequency.

Diffractive multifocal IOLs with fewer diffractive rings and lower add power could theoretically improve the quality of vision after cataract surgery. Trifocal IOLs need it split more light energy to form the third focal point compared to bifocal IOLs and more diffractive rings are used for the trifocal IOLs compared to the IOLs used in our study. This might have an effect on the quality of vision for near and distanace [5–7]. Montes-Mico R et al. [5] used optical bench testing to confirm the quality of the apodized trifocal IOL (Finevision Micro F, PhysIOL, Liege, Belgium), and report a worse quality of vision compared bifocal diffractive IOLs. Kohnen T et al. [7] reported that halo and glare appeared in 60% and 28% of patients, respectively, after the implantation of AT LISA tri839MP, another trifocal IOL (Carl Zeiss Meditec, Jena, Germany). Our study showed halo and glare in 31.5% and 5.3% of patients, respectively, less visual artifacts compared to the results of Kohnen et al. Jonker et al. [6] also reported that mesopic contrast sensitivity was slightly decreased in eyes with diffractive trifocal IOLs compared to diffractive bifocal IOLs. Future studies should compare the quality of vision between groups with bilaterally implanted with diffractive trifocal IOLs and contralaterally implanted diffractive bifocal IOLs.

Reading performance, such as reading speed, critical print size, and threshold print size, were significantly improved postoperatively compared to baseline. Alfonso et al. [14] reported critical print size and threshold size after bilateral implantation of AcrySof + 3.0 toric multifocal IOLs were 0.28 ± 0.12 logRAD and 0.08 ± 0.08 logRAD, respectively. Schmickler et al. [23] reported that critical print size was 0.27 ± 0.12 logRAD in patients after bilateral implantation of Tecnis ZMB00 + 4.0 diffractive multifocal IOLs. Our results of critical print size and threshold print size were 0.24 ± 0.13 logRAD and 0.14 ± 0.13 logRAD, respectively, and comparable to previous studies [14, 23]. In our study, postoperative reading speed was 86.83 ± 17.45 wpm. Alfonso et al. [14] reported a reading speed of 132.68 ± 23.69 wpm after the implantation of diffractive multifocal IOLs. Reading speed in our study is slightly lower compared to results from Western regions [14, 24]. One study using the same application as in our study to test reading speed in Koreans reported a reading speed of 129.7 ± 25.9 wpm for adults in their 20s and 30s [13]. Considering that the reading speed of young adults without presbyopia is faster than that of the older adults with presbyopia, it is possible that the difference in the testing method and characteristics of the languages are the reason for the variance between the results [6, 14, 24].

When the overall satisfaction was evaluated on a five-point scale, satisfaction with distance, intermediate and near vision was 4.42 ± 0.51, 4.11 ± 0.81, and 3.89 ± 0.88, respectively. The results showed that most patients were satisfied. When patients were asked if they would choose the same IOLs again and if they would recommend the IOLs to others, 68.4% of the patients (13 of 19) would choose the same IOLs and recommend it to others.

In this study, ocular dominance was tested prior to cataract surgery, and ZKB00 (add power + 2.75D) was implanted in the dominant eyes and ZLB00 (add power + 3.25D) was implanted in the non-dominant eyes. We assumed that the ‘relatively far’ near focus (ZKB00) in the dominant eye and ‘relatively near’ near focus (ZLB00) in the non-dominant eye would benefit according to the classic monovision trial. However, due to conflicting results with cross monovision results, it may be necessary to conduct additional research to compare the results with cross monovision [25]. Although both eyes of each patient were implanted with different add power, visual acuities of the dominant and non-dominant eyes at each distances were not statistically different. This may be due to the fact that difference in add power between the two IOLs was only 0.5 D. When patients were asked whether they could feel differences between eyes, 17 out of 19 patients did not perceive any difference between both eyes and they did not feel uncomfortable with it. It would be interesting to apply the mix-and-match technique using IOLs with an add power of + 2.75 D and + 4.00 D.

The strength of this prospective contralateral study is the first study applying bilateral mix-and-match implantation of Tecnis multifocal IOLs. Second strength is visual acuities were measured at 6 different distances and that an objective measure of the expected vision at different distances was performed with a defocus curve. Previous studies measured intermediate and near visual acuity only at a single distance. And we comprehensively evaluate clinical outcomes including reading performance, contrast sensitivity and questionnaire. The limitation of this study is the missing direct comparison with bilaterally implanted IOLs with the same add power. However, the results of defocus curve of this study were good and not inferior to those of previous studies.

## Conclusions

In conclusion, the mix-and-match technique using Tecnis multifocal IOLs with low add power is an effective way to achieve good visual acuity over a wide range without affecting quality of vision. The mix-and-match technique is an interesting option for patients who want to be spectacle-free after cataract surgery.

## Abbreviations

CDVA: Corrected distance visual acuity
IOL: Intraocular lens
logMAR: Logarithm of the minimal angle of resolution
logRAD: Logarithm of the reading acuity determination
MR: Manifest refraction
UDVA: Uncorrected distance visual acuity
UIVA: Uncorrected intermediate visual acuity
UNVA: Uncorrected near visual acuity
WPM: Words per minute
